# Hypertension and Social Capital in Indonesia

**DOI:** 10.3390/ijerph23050621

**Published:** 2026-05-08

**Authors:** Younoh Kim, Vlad Radoias

**Affiliations:** Department of Economics and International Business, Sam Houston State University, Huntsville, TX 77341, USA

**Keywords:** social capital, community participation, hypertension, blood pressure, developing countries, Indonesia

## Abstract

**Highlights:**

**Public Health Relevance—How does this work relate to a public health issue?**

- Hypertension is an important public health concern in developing nations, such as Indonesia.
- This study links participation in community programs to cardiovascular health in Indonesia.

**Public Health Significance—Why is this work of significance to public health?**

- We show significant associations between community participation and lower blood pressure.
- We demonstrate how social capital can have a protective effect against hypertension in resource-constrained countries.

**Public Health Implications—What are the key implications or messages for practitioners, policy makers and/or researchers in public health?**

- Fostering community based programs can be a viable strategy to improve health outcomes.
- Policymakers should integrate social capital into public health initiatives.

**Abstract:**

Hypertension is a serious public health concern in developing countries, where the lack of resources and the poor infrastructure often lead to a high incidence of disease and also to high rates of underdiagnosis. We study the linkages between hypertension and social capital in Indonesia. Indonesia is a large developing economy, where social capital is encouraged and plays a significant role. We use participation in community programs as a proxy for social capital, specifically capturing the structural dimension of social networks. By engaging in these programs, individuals create the social ties that facilitate resource sharing and gain access to socially embedded resources. We find significant associations between participating in community programs and having lower blood pressure and lower likelihood of hypertension. These results underscore the public health significance of social capital as a potential non-clinical determinant of health. From a policy perspective, the findings suggest that strengthening community-based social infrastructure could offer an alternative strategy to mitigate hypertension in resource-constrained economies.

## 1. Introduction

Hypertension is a major public health problem across the world. Developing countries in particular face a rising risk associated with the fact that hypertension can be asymptomatic and can go unnoticed for years, in the absence of routine medical screenings. Due to a lack of resources and ease of access to medical services, such routine screenings are very rare in developing countries. Despite a lack of apparent symptoms, hypertension can have devastating health consequences. It is a major risk for coronary heart disease and can lead to stroke and organ damage [1].

Indonesia is the fourth most populous country in the world and has seen tremendous development over the past decades but continues to struggle with a series of public health issues. There is rapid population aging and an increasing old-age dependency ratio [2], an alarming increase in stroke, diabetes, and hypertension [3], and extremely high rates of smoking [4]. Of significant interest to us is the high incidence of hypertension and extremely high rates of hypertensive patients who are not even aware of it. Specifically, 67% of the men and 54% of the women who were found to be hypertensive in a large national survey had never been previously diagnosed by a doctor [5,6].

Our paper aims to study the linkages between hypertension and social capital in Indonesia. Social capital, as proposed by Putnam [7], includes things like community trust, community participation, and norms of reciprocity. It can be a significant resource, equally important to physical and human capital, for the individual and society. There is a rich literature finding strong associations between different measures of social capital and human health [8,9,10]. A lot of this literature, however, is focused on developed countries, which have completely different social environments from those of developing countries. Developing countries also face different constraints and challenges, such as poor infrastructure and a lack of a universal social security system.

The literature on social capital and health in Indonesia is almost non-existent, with only a handful of papers that mainly focus on mental health and aging [11,12,13] and intergenerational effects between mothers’ social capital and children’s health [14,15]. A more recent paper by Kim and Radoias [16] also looks at measures of physical health but does not consider hypertension specifically. We could only find one previous study that looks at hypertension. Cao and Rammohan [17] consider hypertension in their analysis but find no significant associations between social capital and hypertension. Their study has several significant shortcomings, however. First and foremost, they use a prior hypertension diagnosis as their main indicator of disease, and not actual blood pressure measurements. As mentioned before, Indonesia is faced with a significant percentage of its population not being properly diagnosed. Secondly, their study only uses a sub-sample of a nationally representative data set, when a much larger dataset is in fact available. We address both shortcomings in our study and find that there are, in fact, significant linkages between social capital and hypertension.

Studying these linkages in the Indonesian context is important for several reasons. Studies such as Shiell, Hawe, and Kavanagh [18] show that social capital interventions are extremely context-specific, so results from other countries may not directly translate to the Indonesian context. At the same time, while a large set of the literature points to the positive health effects of social capital, there are also studies documenting negative consequences of social capital [19]. Therefore, whether social capital correlates positively or negatively with hypertension in Indonesia cannot be inferred from extrapolating the results from other countries.

Lastly, social capital is at the forefront of public policy in Indonesia. Since the public health infrastructure in Indonesia is generally poor and there is no universal social safety net, the population relies heavily on family and social groups. This is especially important at older ages and in rural settings. There are different types of social participation programs available and encouraged by local governments, such as voluntary labor, religious activities, and community credit programs [13]. Therefore, Indonesia provides a compelling case study due to its unique combination of robust community-based programs and prevalent public health challenges, presenting researchers with rich socio-economic data for analyzing the linkages between social capital and human health in a developing economy.

The link between community participation and hypertension could theoretically be explained through several potential mechanisms. Community participation specifically captures the structural dimension of social capital. High levels of social ties could act as a stress buffer, reducing cortisol levels and cardiovascular reactivity. Furthermore, participating in community programs could facilitate the flow of health-related information among participants and strengthen the norms of mutual aid, which may further improve health-seeking behavior among hypertensive individuals.

## 2. Data and Methodology

We employ a cross-sectional analysis using data from the 5th wave of the Indonesian Family Life Survey (IFLS). IFLS is a high-quality longitudinal survey that is representative of 83% of the Indonesian population. While the survey spans five main waves (1993–2015), this study focuses on the 5th wave to capture the most recent snapshot of the issue under consideration. The IFLS contains a variety of measures that address socio-economic and health factors that are important for our investigation.

We collect data on structural social capital, which is proxied by a series of questions regarding different social institutions and programs existing in the community and respondents’ level of participation in these programs. More specifically, we aggregate respondents’ answers to a series of 5 questions regarding participation in community meetings, voluntary labor programs, community improvement programs, religious activities, and arisan, which is a form of rotating credit program very common in Indonesia. Each question was first assigned an indicator variable coded as 1 if the respondents participated in the program during the last 12 months, and 0 otherwise. We then added all these individual indicator variables into one aggregate measure of social capital. It is important to note that a 0 could mean either voluntary non-participation or non-participation due to lack of access if a particular activity is not available in a community. In either case, the respondent does not benefit from access to social capital structures.

While we recognize that various aggregation methods exist, we believe that an equally weighted index of the five individual participation variables collected by IFLS is the most transparent and conservative approach in this context. In the absence of a dominant theoretical framework or previous empirical results on the issue, assigning specific weights to different activities could inadvertently introduce researcher bias.

For our outcome hypertension measures, we use the actual blood pressure data collected in IFLS. Trained nurses took blood pressure measurements for all IFLS respondents older than 15. There are a total of three blood pressure measurements taken for each respondent. To alleviate measurement errors due to respondents being nervous, we dropped the first measurement and then averaged the last two measurements to get the main systolic and diastolic blood pressure outcome variables. This approach is a standard epidemiological practice designed to reduce measurement error and minimize the potential for “white coat” hypertension [20,21]. We also constructed a third outcome variable, an indicator for elevated blood pressure status, which we defined according to the WHO definition of being hypertensive (higher than 140 mm Hg systolic/90 mm Hg diastolic).

It is important to note that, given the significant underdiagnosis of hypertension in Indonesia, we purposefully define our elevated blood pressure status variable based purely on actual physiological measurements taken during IFLS, rather than considering formal clinical histories of the respondents. We acknowledge that this approach classifies individuals with medically managed hypertension as non-hypertensive if they exhibit normal blood pressure readings. This classification is consistent with our objective to identify associations between social capital and active elevated blood pressure. If social participation facilitates medical access or adherence that successfully brings a clinically hypertensive patient into a normal blood pressure range, we consider this a realized health benefit of social capital. Conversely, labeling successfully managed patients as hypertensive based solely on their clinical diagnosis and despite normal blood pressure readings could introduce a downward bias, potentially masking the very mechanism through which social capital improves population health.

Formally, the cross-sectional econometric model we estimate is of the form:

*H_i_* = *αCommunityParticipation_i_* + *βX_i_* + *ε_i_*

where *H_i_* is the health outcome of respondent *i* and *X_i_* is the vector of control variables for respondent *i*. The parameter of interest is *α*, which measure the effect of social capital on the health outcome.

As control variables, we use respondents’ age and age squared (to control for non-linearities), years of education, per capita household expenditure (PCE), an indicator for being overweight (coded with 1 for respondents having a BMI over 25), urban/rural location, a gender indicator, overall general health status (GHS), and subjective social status (SSS).

Subjective Social Status (SSS) is constructed based on respondents’ perceived standing on a six-rung income ladder. We coded High SSS as 1 for respondents who perceived themselves on the highest three rungs of the ladder and 0 otherwise. While alternative construction methods for an SSS indicator may be employed, we followed an approach consistent with the extant literature, where SSS has been found to be an important predictor of health, even after controlling for objective socio-economic conditions [4,22].

General Health Status (GHS) is a self-reported measure of health that proxies for a variety of health conditions. Respondents were asked to score their general health as either very healthy, somewhat healthy, somewhat unhealthy, or unhealthy. We coded a Poor GHS indicator as 1 for all respondents who rated their health as somewhat unhealthy or unhealthy and 0 otherwise. Similarly constructed GHS indicators have been found to be good predictors of future health and are commonly used in socio-economic research to proxy for the overall level of health of the individual.

Our sample contains over twenty-three thousand respondents, which ensures high levels of statistical significance. To deal with missing data, we used listwise deletion, so this sample was obtained after dropping the IFLS respondents who were missing data for any of the variables we use in our empirical specification. There were 34,464 initial respondents in the original IFLS sample (B3A_DL1 module). A total of 2149 of these respondents were dropped for missing blood pressure data and 9137 more respondents were further dropped for missing data on covariates. The final remaining sample that we analyze contains 23,178 respondents with full information on the entire set of variables.

While the large sample size of our remaining sample protects the statistical power of our estimates, we acknowledge that this method could potentially lead to bias if the data are not missing completely at random and the missingness is correlated with our key variables of interest. To investigate the possibility and the nature of this bias, we perform several *t*-tests to check whether the means of the relevant variables for the included and excluded groups are statistically different. These tests confirm that the data is not missing completely at random, which leads to selection bias. Specifically, the respondents who are excluded due to missing data show slightly higher blood pressure measurements than the respondents who are included in the final sample (mean systolic 128.28 for the missing group vs. 127.07 for the included group). The missing group also shows slightly lower community participation than the included group (mean 1.49 for the missing group vs. 1.65 for the included group). These differences are statistically significant, but modest in their magnitudes, so the bias introduced is arguably also modest in size. Furthermore, under a stable functional form assumption, the sign of these correlations suggests a likely attenuation bias and therefore an underestimation of the true correlations. If data was not missing for the excluded group, the estimated correlations would likely be stronger. Our estimates are therefore conservative in their magnitude.

Furthermore, we recognize minor inconsistencies in the age variable for a small subset of the sample. Specifically, one observation reports an age of 7 and 20 observations report an age of 14, despite blood pressure measurements typically being restricted to respondents aged 15 and older. These likely represent survey artifacts or minor discrepancies in the timing between different survey modules. To ensure the integrity of our findings, we conducted a sensitivity check by excluding these 21 observations. Their removal does not substantively alter our coefficients or significance levels, confirming that our results are robust to these minor sample inconsistencies. Table 1 below presents some basic descriptive statistics of our sample:

It is difficult to hypothesize theoretically about the sign of the correlations between social capital and blood pressure. The social capital literature is very scarce in the Indonesian context, and studies using data from other countries may not possess external validity. Furthermore, even when considering this literature, the empirical evidence collected from other countries is not unanimous. Social capital, especially when proxied by community participation, has both positive and negative effects on health. On one hand, community participation may lower arterial blood pressure through channels such as improved sense of belonging, increased level of physical activity, reduction in stress, promotion of healthy habits, and access to community credit and finance. On the other hand, social capital can increase arterial blood pressure through the social contagion of risky behaviors such as smoking and unhealthy diets or through the burden of participation imposed on citizens who may otherwise feel marginalized in their community. Given these opposite effects, which make theoretically assessing these correlations impossible, it is important to assess these effects empirically, using nationally representative data and robust measures of blood pressure, which is where our study is making its most important contribution.

## 3. Results

Table 2 presents the results of our main estimations. Column 1 presents the associations between social capital and systolic blood pressure, column 2 presents the associations with diastolic blood pressure, and column 3 presents the associations with elevated blood pressure status.

The results are consistent and statistically significant. Social capital is associated with having lower systolic blood pressure, lower diastolic blood pressure, and a lower likelihood of having elevated blood pressure. While the results of these estimations cannot be interpreted as causal, these associations are highly suggestive of the effect that community participation may have on hypertension, since we control for a significant number of socio-economic factors and hypertension risk factors.

While the estimated effect size per individual may appear modest, the practical significance is substantial when extrapolated to the population level. A marginal reduction in mean systolic blood pressure across Indonesia’s population of over 270 million would result in a significant decrease in the aggregate incidence of stroke and cardiovascular events, potentially reducing the national burden on healthcare infrastructure.

Since IFLS uses a multi-stage stratified sampling design, individuals in the same community may share unobserved characteristics. To account for potential intra-cluster correlation that could deflate standard errors and lead to artificially low *p*-values, we re-estimated the econometric models using standard errors clustered at the community level. The results remained robust, with only the coefficient on diastolic blood pressure slightly dropping in significance from the 1% significance level to slightly under 2% (*p*-value of 0.018). The other two coefficients of importance preserved their 1% levels of statistical significance even when adjusting for clustering. These results are included in Table A1 in Appendix A.

It is important to acknowledge and discuss the potential limitations of this cross-sectional study. Specifically, the presence of unobserved community characteristics or confounders such as smoking, dietary habits, levels of physical activity, comorbidities not captured by GHS, and use of certain medications may bias the estimated associations. Nevertheless, these findings offer substantial value as they provide some of the first empirical evidence linking structural social capital to objective health biomarkers in the Indonesian context. While these associations are not strictly causal, they establish a baseline for understanding how community engagement may serve as a protective factor against chronic disease in a large, developing nation. Future research using longitudinal data will be essential to further isolate these causal pathways and address potential sources of bias.

It is also important to consider that participation in community programs may yield both positive and negative effects. While engagement can increase physical activity, reduce stress, and promote healthy lifestyle choices, the “dark side” of social capital is well documented in the literature (Villalonga-Olives and Kawachi, 2017) [19]. For instance, social contagion mechanisms can propagate risky health behaviors as effectively as healthy ones. Furthermore, while community involvement serves as a stress buffer for many, it may inadvertently increase stress for others due to social obligations or group conflict. Our findings suggest that in the Indonesian context, the beneficial effects appear to outweigh the potential negatives; however, further research is needed to disentangle these divergent channels, identify the specific conditions under which community participation becomes most protective, and also verify causality.

## 4. Conclusions

Our results demonstrate statistically significant associations between social capital, as proxied by participation in community programs, and blood pressure. Specifically, participation in community programs is associated with lower blood pressure and a decreased probability of hypertension. These results are robust to the inclusion of a series of socio-economic and health risk factors. The statistical significance of these associations is also robust to community-based clustering.

From a policymaking perspective, these findings are important given that hypertension is a severe issue in Indonesia. While community participation is highly encouraged, a previous study using lower-quality data found no such correlations. We show that significant associations do exist when using higher-quality data. This highlights the potential importance of social capital, especially in developing countries like Indonesia, where the public health infrastructure may not yet be fully equipped to address the high incidence of chronic disease.

At the same time, we stress that community participation should not be viewed as an absolute positive, as it may also have unintended negative consequences for other public health concerns. More research needs to be done to establish true causal links and to fully understand and disentangle the positive and negative channels through which community participation could affect human health. In the specific context of hypertension, the positives appear to outweigh the negatives, at least within the scope of the associations observed in our analysis. A better understanding of the effects at work may lead to even greater net benefits if these community programs are optimized in such a way as to maximize the positives and minimize the negatives.

Our findings suggest that fostering structural social capital, if this is confirmed to have a true causal link by future research, could be a viable, low-cost strategy for managing the growing burden of hypertension in Indonesia. Public health departments and policymakers should prioritize the integration of routine blood pressure screenings into existing community-based programs or local religious and neighborhood gatherings. By taking advantage of these established social networks, policymakers can implement silent interventions such as health education or exercise initiatives that normalize cardiovascular wellness without the need for extensive and expensive clinical infrastructure. Furthermore, such programs should be designed to maximize participation while monitoring for potential “dark side” effects, ensuring that social obligations do not inadvertently increase stress for vulnerable participants. Ultimately, investing in the social fabric of communities could serve as a critical non-clinical determinant of health that can complement formal medical services in developing economies.

## Figures and Tables

**Table 1:** Summary Statistics.

Variable	Minimum	Maximum	Mean	Std. Dev.
Systolic Blood Pressure (mm Hg)	75.5	240.5	127.07	19.87
Diastolic Blood Pressure (mm Hg)	35	148.5	78.48	11.56
Elevated Blood Pressure Status	0	1	0.2258	0.42
Community Participation	0	5	1.65	1.32
Age (years)	7	101	37.91	14.76
Education Level (years)	0	24	8.86	4.52
Log PCE	11.19	21.93	13.72	0.70
Overweight Indicator	0	1	0.32	0.47
Urban Location Indicator	0	1	0.58	0.49
Male Indicator	0	1	0.49	0.5
Poor GHS Indicator	0	1	0.21	0.41
High SSS Indicator	0	1	0.28	0.45

Sample Size: *N* = 23,178.

**Table 2:** Associations between social capital and blood pressure outcomes.

Dependent Variable Independent Variables	Systolic BP	Diastolic BP	Elevated BP Status
Community Participation	−0.2949 ***	−0.1476 ***	−0.0065 ***
	(0.0886)	(0.0559)	(0.0020)
Age	−0.05897	0.5428 ***	−0.0002
	(0.0429)	(0.0238)	(0.0009)
Age Squared	0.0076 ***	−0.0039 ***	0.0001 ***
	(0.0005)	(0.00029)	(0.00001)
Overweight	8.5535 ***	6.0371 ***	0.1522 ***
	(0.2618)	(0.1637)	(0.0061)
Years of Education	−0.3364 ***	−0.1004 ***	−0.0042 ***
	(0.0309)	(0.0189)	(0.0007)
Log PCE	0.2331	0.1567	0.0085 **
	(0.1732)	(0.1070)	(0.0039)
Urban	−0.1155	0.3719 **	0.0099 *
	(0.2332)	(0.1454)	(0.0052)
Male	5.5849 ***	1.5271 ***	0.0329 ***
	(0.226)	(0.1436)	(0.0051)
Poor GHS	0.8426 ***	0.4435 **	0.0189 ***
	(0.3017)	(0.182)	(0.0064)
High SSS	−0.062	0.1122	0.0101 *
	(0.2501)	(0.1583)	(0.0057)
Constant	111.42 ***	60.30 ***	−0.1186 **
	(2.3954)	(1.465)	(0.0535)
Sample Size	23178	23178	23178

Heteroskedasticity-robust standard errors in parentheses. ***—statistically significant at the 1% level. **—statistically significant at the 5% level. *—statistically significant at the 10% level. Column 3 (Elevated BP Status) was estimated using a linear probability model.

## Data Availability

The IFLS data used in this study are publicly available at https://www.rand.org/health/surveys/FLS/IFLS.html.

## Appendix A

**Table A1:** Associations between social capital and blood pressure outcomes.

Dependent Variable Independent Variables	Systolic BP	Diastolic BP	Elevated BP Status
Community Participation	−0.2949 ***	−0.1476 **	−0.0065 ***
	(0.0974)	(0.0623)	(0.0022)
Age	−0.05897	0.5428 ***	−0.0002
	(0.0464)	(0.0249)	(0.0009)
Age Squared	0.0076 ***	−0.0039 ***	0.0001 ***
	(0.0006)	(0.00031)	(0.00001)
Overweight	8.5535 ***	6.0371 ***	0.1522 ***
	(0.2744)	(0.16653)	(0.0066)
Years of Education	−0.3364 ***	−0.1004 ***	−0.0042 ***
	(0.0334)	(0.0198)	(0.00075)
Log PCE	0.2331	0.1567	0.0085 **
	(0.1893)	(0.12297)	(0.00396)
Urban	−0.1155	0.3719	0.0099
	(0.35965)	(0.2328)	(0.0071)
Male	5.5849 ***	1.5271 ***	0.0329 ***
	(0.2279)	(0.1487)	(0.0054)
Poor GHS	0.8426 ***	0.4435 **	0.0189 ***
	(0.3071)	(0.1828)	(0.00695)
High SSS	−0.062	0.1122	0.0101 *
	(0.2586)	(0.1637)	(0.0057)
Constant	111.42 ***	60.30 ***	−0.1186 **
	(2.6927)	(1.6962)	(0.0548)
Sample Size	23178	23178	23178

Clustered standard errors in parentheses (community level clustering). ***—statistically significant at the 1% level. **—statistically significant at the 5% level. *—statistically significant at the 10% level. Column 3 (Elevated BP Status) was estimated using a linear probability model.
